# Rac1/WAVE2 and Cdc42/N-WASP Participation in Actin-Dependent Host Cell Invasion by Extracellular Amastigotes of *Trypanosoma cruzi*

**DOI:** 10.3389/fmicb.2018.00360

**Published:** 2018-02-28

**Authors:** Alexis Bonfim-Melo, Éden R. Ferreira, Renato A. Mortara

**Affiliations:** Departamento de Microbiologia, Imunologia e Parasitologia, Escola Paulista de Medicina, Universidade Federal de São Paulo, São Paulo, Brazil

**Keywords:** *Trypanosoma cruzi*, extracellular amastigotes, Rho GTPases, actin, host cell invasion

## Abstract

This study evaluated the participation of host cell Rho-family GTPases and their effector proteins in the actin-dependent invasion by *Trypanosoma cruzi* extracellular amastigotes (EAs). We observed that all proteins were recruited and colocalized with actin at EA invasion sites in live or fixed cells. EA internalization was inhibited in cells depleted in Rac1, N-WASP, and WAVE2. Time-lapse experiments with Rac1, N-WASP and WAVE2 depleted cells revealed that EA internalization kinetics is delayed even though no differences were observed in the proportion of EA-induced actin recruitment in these groups. Overexpression of constitutively active constructs of Rac1 and RhoA altered the morphology of actin recruitments to EA invasion sites. Additionally, EA internalization was increased in cells overexpressing CA-Rac1 but inhibited in cells overexpressing CA-RhoA. WT-Cdc42 expression increased EA internalization, but curiously, CA-Cdc42 inhibited it. Altogether, these results corroborate the hypothesis of EA internalization in non-phagocytic cells by a phagocytosis-like mechanism and present Rac1 as the key Rho-family GTPase in this process.

## Introduction

*Trypanosoma cruzi* is a protozoan parasite that causes Chagas' disease and affects approximately 6–7 million people worldwide, mostly in Latin America (WHO., 2017). Classically, infection begins by metacyclic trypomastigote forms released in the feces of triatomine vectors. Unlike the metacyclic or bloodstream trypomastigote forms, host cell invasion by extracellular amastigotes (EAs) is highly dependent on the actin cytoskeleton of host cell (Mortara et al., 2005; Ferreira et al., 2012).

During host cell invasion, EAs induce recruitment and colocalization with actin of diverse host cell molecules, such as integrins, extracellular matrix components and actin binding proteins, in a cup-like structure (Procópio et al., 1999). EAs also promote the sequential and coordinated formation of phosphoinositides at their entry site on the plasma membrane of HeLa cells, suggesting that they induce a phagocytosis-like process in non-phagocytic cells (Fernandes et al., 2013). Recently, our group also showed that EAs induce selective phosphorylation of cortactin by ERK, which is abolished if heat-killed parasites or non-infective epimastigote forms are used (Bonfim-Melo et al., 2015). These studies demonstrate the importance of the actin cytoskeleton and its regulatory proteins during EA invasion of non-phagocytic cells.

Cdc42, Rac1, and RhoA, the key regulators of actin cytoskeleton signaling, have been evaluated during invasion of intracellular bacteria, viruses and protozoa (Krause-Gruszczynska et al., 2011; Reed et al., 2012; Van den Broeke et al., 2014). Cdc42 and Rac1 induce actin polymerization by the Arp2/3 complex through binding to and activation of their effector proteins, N-WASP and WAVE-2, respectively (Spiering and Hodgson, 2011). In canonic phagocytosis, actin polymerization is mediated by these proteins during their translocation to the plasma membrane after formation of phosphatidylinositol bi (4,5) or tri (3,4,5) phosphate (PIP_2_ or PIP_3_) at the inner leaflet of the plasma membrane (Takenawa and Suetsugu, 2007; Spiering and Hodgson, 2011). During actin remodeling, signaling of Rho GTPases can cooperate or inhibit each other's activity (Guilluy et al., 2011). For instance, RhoA effector protein ROCK is able to activate FilGAP, a Rac1 inhibitory protein (Ohta et al., 2006). Rac1 activity can also be inhibited after the recruitment of PBR (polybasic region) containing GAPs (GTPase Activating Proteins) by PIP_3_ generated after activation of PI3k by Cdc42 (Campa et al., 2015). N-WASP and WAVE2 pathways can cooperate or not during invasion of *Listeria* ssp. depending on the host cell (Bierne et al., 2005). Using MDCK cells stably expressing Rho GTPase constructs, our group showed that Rac1 is involved in G strain EAs invasion but not invasions by other parasite strains or forms (Fernandes and Mortara, 2004). Despite these initial results, their precise role during *T. cruzi* internalization remains poorly characterized.

Considering actin involvement in EA internalization and the importance of Rho-family GTPases in actin dynamics, the aim of this study was to evaluate the role of Rho GTPases and their effector proteins, N-WASP and WAVE-2, in microfilament modulation during host cell invasion by EAs. Using cells depleted of or overexpressing these proteins and microcopy techniques, we found that Rac1 is the key Rho GTPase in this process, possibly acting together with WAVE2, whereas Cdc42 displays a minor role in parallel with N-WASP participation; RhoA had a negative role in the regulation of actin dynamics involved in EA entry.

## Materials and methods

### Antibodies, reagents and plasmids

Mouse anti-Rac1 (clone 23A8, #05-389) and rabbit anti-Cdc42 (#07-1466) were purchased from Millipore. Rabbit anti-RhoA (R9404) and mouse anti-WAVE2 (clone 8E7, WH0010163 M2) were from Sigma-Aldrich. Rabbit anti-N-WASP (clone 30D10, #4848), mouse β-actin (clone 8H10D10, #3700) and rabbit anti-GAPDH (clone D16H11, #5174) were from Cell Signaling. Mouse anti-c-myc (clone 9E10, MA1-980) was from Invitrogen. Secondary antibody goat anti-rabbit Alexa Fluor 488 (A11008) was from Invitrogen. Goat anti-rabbit IgG-peroxidase (A6154), goat anti-mouse IgG-peroxidase (A4416) and goat anti-human Alexa Flour 488 (A11013) were from Sigma-Aldrich. Anti-*T. cruzi* was previously used (Mortara et al., 1999).

DAPI (4′,6-diamidino-2-phenylindole, 01306) and Hoechst 33342 (H1399) were from Invitrogen. Phalloidin-TRITC (P1951), puromycin (P8833) and polybrene (107689) were from Sigma-Aldrich. FuGene HD (04709705001) was from Roche. Protein Assay Dye Reagent Concentrate (#500-0006) was obtained from BioRad.

Plasmids containing WT, DN or CA constructs of Cdc42 or Rac1 were made by Dr. Klaus Hahn (University of North Carolina, EUA; Kraynov et al., 2000; Nalbant et al., 2004; Machacek et al., 2009) and obtained from the Addgene repository (#12599, #12601, #12600, #13719, #13721, #13720, respectively). Plasmids containing WT, DN or CA constructs of RhoA were made by Dr. Alan Hall (Memorial Sloan Kettering Cancer Center, USA; Nobes and Hall, 1999) and obtained from Addgene (#15899, #15901, #15900, respectively). N-WASP-GFP construct was kindly provided by Dr. Stéphane Gasman (Université de Strasbourg, France; Moreau et al., 2000). WAVE2-YFP construct was kindly provided by Dr. Daniel Billadeau (Mayo Clinic, USA; Nolz et al., 2008). LifeAct-RFP was purchased from Ibidi (60102). ShRNAi sequences were provided in pLKO backbone and purchased from Sigma-Aldrich: Cdc42/NM_001791 (TRCN0000299931: CCTGATATCCTACACAACAAA and TRCN0000299932: CAGATGTATTTCTAGTCTGTT), Rac1/NM_006908 (TRCN0000318375: CCCTACTGTCTTTGACAATTA and TRCN0000318430: GCTAAGGAGATTGGTGCTGTA), RhoA/NM_001664 (TRCN0000047710: GTACATGGAGTGTTCAGCAAA and TRCN0000047711: TGGAAAGACATGCTTGCTCAT), N-WASP/NM_003941 (TRCN0000123061: GCACAACTTAAAGACAGAGAA and TRCN0000123062: CAGGAAACAAAGCAGCTCTTT), WAVE2/NM_006990 (TRCN0000379527: CAGACCCTTCATACTTCTTTG and TRCN0000379885: CCACTTTGGTGTACCAGAATG) and scramble/Scr (SHC002: CAACAAGATGAAGAGCACCAA). psPAX2 was a gift from Didier Trono (Addgene plasmid # 12260). pCMV-VSV-G was a gift from Bob Weinberg (Addgene plasmid # 8454) (Stewart et al., 2003).

### Cell cultures and establishment of depleted cell lineages

G strain of *T. cruzi* [*T. cruzi* I, capable to infect humans (Zingales, 2017)] was used throughout this work (Yoshida, 1983) and was manipulated in level 2 biosafety cabinets following institutional safety procedures. EAs were generated by 14 h incubation of trypomastigotes derived from Vero cells tissue cultures (TCT) in liver infusion tryptose (LIT) medium at 5.8 pH and 10% of fetal bovine serum (FBS) (Ley et al., 1988; Bonfim-Melo et al., 2015). Parasites were incubated in complete RPMI medium at pH 7.3 for 1 h prior to each experiment.

HeLa (human cervical adenocarcinoma cells) and Vero (green monkey epithelial kidney cells) cells were obtained from Instituto Adolfo Lutz (São Paulo, Brazil). Both cell lines were kept in RPMI medium with 10% FBS supplemented with antibiotics (10 μg/mL streptomycin and 100 U/mL penicillin, Sigma-Aldrich, St. Louis, MO, USA) in a humidified atmosphere at 37°C with 5% CO_2_. HEK293T (human kidney embryonic cells, kindly provided by Dr. Silvia Boscardin from ICB-USP) were cultivated in DMEM (Dulbecco's Modified Essential Medium, Sigma-Aldrich) with 10% FCS and antibiotics (10 μg/mL streptomycin and 100 U/mL penicillin, Sigma-Aldrich, St. Louis, MO, USA) in a humidified atmosphere at 37°C and 5% CO_2_.

### Lentiviral transduction and HeLa lineage establishment

HEK293T cells (5 × 10^6^) were plated in 10-cm diameter plates with complete medium. Twenty-four hours later, cells were transfected by calcium phosphate precipitation with psPAX2 and pCMV-VSV-G along with the different pLKO plasmids described above. Six hours later, the medium was removed, and cells were incubated for 2 min with 15% glycerol (in PBS), then washed twice with PBS. Complete DMEM medium was added. After 24 and 48 h, supernatant was collected, filtered with 0.45-μm pore filters and stocked at −80°C. A suspension with 2 × 10^4^ HeLa cells was plated per well in a 6-well plate with complete medium. After 24 h, 2 mL of lentivirus were added to each well in the presence of 8 μg/mL polybrene, and after 24 h they were supplemented with an additional 2 mL of complete medium. Forty-eight hours post-transduction, selection of cells transduced and expressing shRNAis sequences was started with increasing concentrations of puromycin, beginning with 0.2 μg/mL and up to 10 μg/mL after 2 weeks. Protein depletion was evaluated by densitometry by ImageJ (NIH) and western blotting following standard protocols (Bonfim-Melo et al., 2015) using the antibodies described herein.

### Immunofluorescence and recruitment assays

For recruitment of GFP/YFP/c-myc tagged proteins, 2 × 10^5^ HeLa cells were plated on 4 coverslips in 6-well plates, and after 24 h, they were transfected with 2–5 μg of plasmids and 6 μL of FuGene HD (Promega) following the manufacturer's instructions. After 9 h, medium was replaced and experiments were conducted 48 h post-transfection. Coverslips containing cells were transferred to 24-well plates, and EAs were added (MOI 10) for 1 h. After washing and fixing, cells were stained with DAPI and phalloidin-TRITC (Sigma-Aldrich) and mounted in pH 9.0 buffered glycerol solution with 1 mM *p*-phenylenediamine as previously described (Platt and Michael, 1983; Bonfim-Melo et al., 2015). EA-induced protein recruitment was evaluated by epifluorescence or confocal microscopy. For recruitment of actin along with GFP/YFP/c-myc tagged proteins, interactions were evaluated under confocal microscopy (TCS SP5 II Tandem Scanner, Leica). Confocal images were processed and rendered with Imaris 7.0.0 (Bitplane) and ImageJ (NIH) software.

For actin recruitment quantification experiments, 3 × 10^4^ depleted HeLa cells were plated in 24-well plates. After 48 h, EA (MOI 10) were incubated for 1 h and cells were washed, fixed and stained with anti-*T. cruzi* serum, phalloidin-TRITC and DAPI as described herein. Actin recruitment induced by EA in depleted HeLa cells was quantified under epifluorescence microscopy (BX51, Olympus). EA interaction quantifications were made with at least 50 HeLa cells in triplicate experiments. Images were analyzed with ImageJ (NIH).

### Confocal microscopy and live cell time-lapse assays

For recruitment of GFP tagged GTPases, 3 × 10^4^ wild type HeLa cells were plated in Hi-Q4 (ibidi) plates, and after 24 h, they were transfected with 2 μg of LifeActin-RFP and 2 μg of Rac1-GFP or Cdc42-GFP with 6 μL of FuGene HD following the manufacturer's instructions. On the next day, medium was replaced and 48 or 72 h post-transfection, cells and parasites (EAs, MOI 10) were stained with Hoechst and interactions were evaluated by time-lapse confocal microscopy. Cells were kept at 37°C with 5% CO_2_ in a humid atmosphere. Five focal planes were acquired (0.7-μm thickness and 2-μm z-step) with 2 min time intervals with 63 × and 1.40 N.A. objective. At least four fields were evaluated in three independent experiments. Confocal images were processed and rendered with Imaris 7.0.0 (Bitplane) and ImageJ (NIH).

For actin recruitment in depleted HeLa cells, cells were only transfected with LifeAct-RFP and all other parameters and protocols remained unaltered.

### Invasion assays

For invasion assays, 1.5 × 10^5^ depleted HeLa cells were plated over coverslips in 24-well plates. After 24 h, EAs were added (MOI 10) for 2 h, the coverslips were fixed with Bouin, stained with Giemsa, and destained by acetone/xylene gradients, and internalized EAs were quantified in at least 300 cells/replicate under optical microscopy (Bonfim-Melo et al., 2015). Experiments were conducted in duplicate or triplicate. For invasion assays using cells overexpressing mutated constructs, 2 × 10^5^ wild type HeLa cells were plated in coverslips in 6-well plates, and they were transfected 24 h later following the protocol described herein. After 9 h, complete medium was replaced. Forty-eight hours post-transfection, coverslips were transferred to 24-well plates and EAs (MOI 10) were incubated for 2 h. Cells were fixed and stained with phalloidin-TRITC and DAPI. Intracellular EAs were quantified in 50 transfected cells/replicate under epifluorescence microscopy. Experiments were repeated at least three times.

### Scanning electron microscopy

To evaluate the morphology of microvilli recruited by EAs, 1.5 × 10^5^ depleted HeLa cells were plated over coverslips in 24-well plates. After 24 h, EA were added (MOI 10) for 1 h and were washed and fixed with 2.5% glutaraldehyde in sodium cacodylate buffer 0.1 M and pH 7.2. After osmium tetroxide 1% impregnation, samples were dehydrated with ethanol gradient solutions and dried by the critical point protocol from CO_2_. Finally, coverslips were glued in stubs sputtered with gold. EA-HeLa cell interactions were observed and documented in a FEI Quanta FEG 250 scanning electron microscope at the Electron Microscopy Center, EPM-UNIFESP.

### Statistical analysis

Results are presented as the mean in dispersion graphs in which each dot represents one replicate from independent experiments. We used One-Way ANOVA test with Tukey's multiple comparison test in Prism 5.0 software (GraphPad), and differences were considered statistically significant when *P* < 0.05.

## Results

### Host cell rho-family GTPases are recruited to actin-rich cup-like structures induced at EA invasion sites

**Figure 1:**
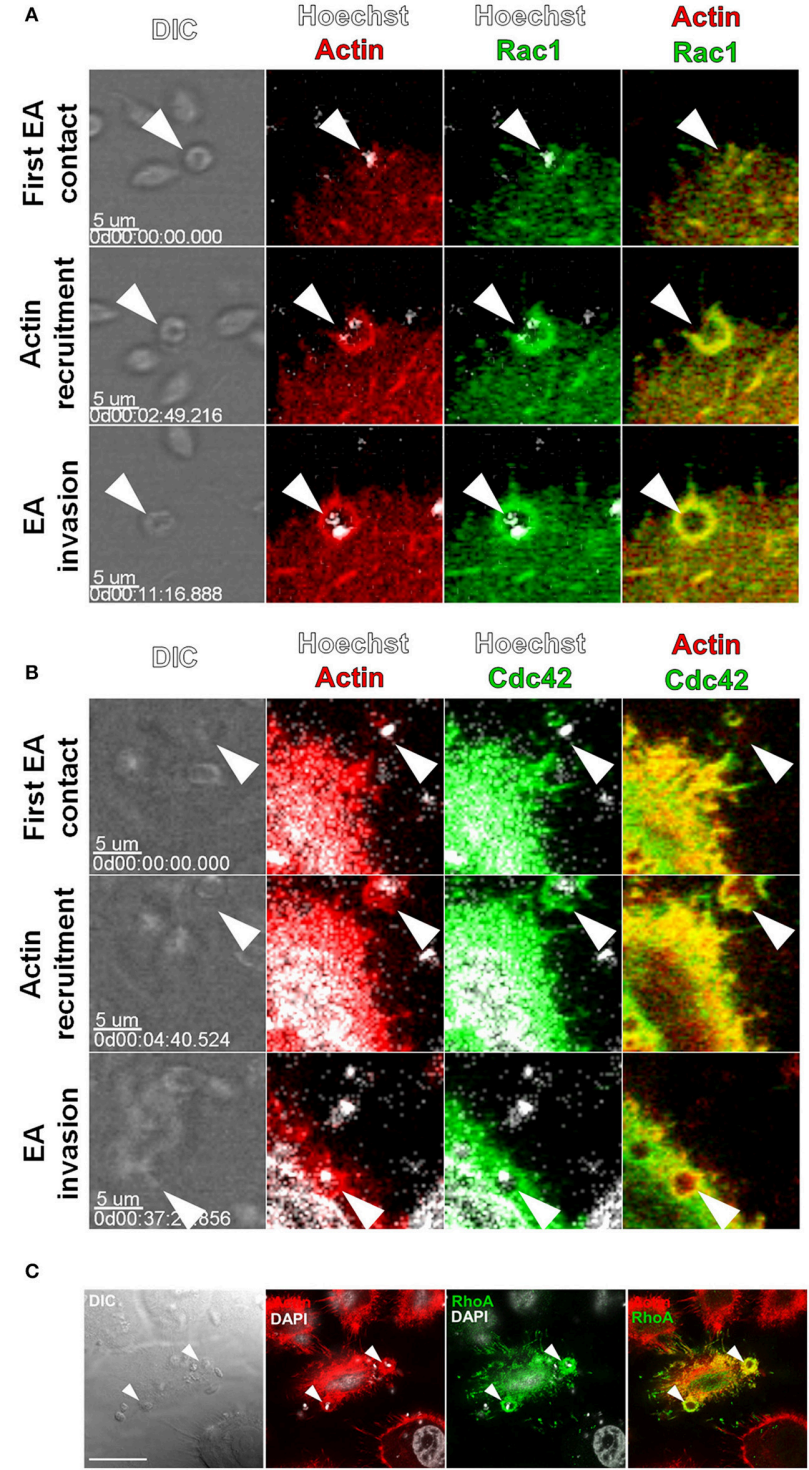
Rac1, Cdc42 and RhoA are recruited to actin-rich cup-like structures induced by EAs. HeLa cells were transfected with Cdc42, Rac1 (GFP tagged) or RhoA (c-myc tagged) and interactions with EAs were evaluated by live confocal microscopy. **(A)** Frames from time-lapse movies showing the moments of initial contact, beginning of actin recruitment and EA (arrowheads) internalization with recruitment and colocalization of Rac1-GFP (green) and LifeAct-RFP (red). **(B)** Frames from time-lapse movies showing Cdc42-GFP (green) recruited along with LifeAct-RFP (red) to EA (arrowheads) invasion sites during internalization. **(C)** Recruitment and colocalization of RhoA-c-myc (green) with actin (red) at EA invasion sites (arrowheads) was observed in fixed cells stained with phalloidin-TRITC. Nuclei and kinetoplasts were stained with Hoechst **(A,B)** or DAPI **(C)**. Bar = 20 μm.

Rac1 and Cdc42 are key GTPases that remodel actin filaments into lamellipodia and filopodia during locomotion and the internalization of intracellular pathogens (Spiering and Hodgson, 2011). We initially cotransfected HeLa cells with GFP tagged Rac1 or Cdc42 and LifeAct-RFP and evaluated their interaction with EAs by time-lapse analysis with confocal microscopy. EAs (Figure 1A, arrowheads) induced recruitment of Rac1-GFP along with actin-RFP to its adhesion/invasion sites with colocalization of both molecules (Figure 1A). Time-lapse experiments revealed that colocalization of both proteins to EA-induced cup-like structures occurred from the initial moment of actin recruitment until parasite internalization. As observed at frame 11′16″ (Figure 1A and Video 1), EA internalization is identified by a decrease in parasite refringence along with the closure of the cup-like structure into a circular-shaped actin staining. Recruitment and colocalization with actin at EA invasion sites was also observed in live cells transfected with Cdc42-GFP (Figure 1B). Also, actin mobilization may last for a few minutes after EA internalization as shown more clearly in the Rac1-GFP interaction (11′16″ in Figure 1A and Video 1) but is less evident in Cdc42-GFP (37′20″ in Figure 1B and Video 2).

RhoA GTPase shares structural features with Rac1 and Cdc42 but mainly promotes the reorganization of actin filaments into stress fibers (Spiering and Hodgson, 2011). EA interactions with HeLa cells expressing RhoA (c-myc tagged) were assessed by confocal microscopy in fixed cells. Similar to Rac1 and Cdc42, RhoA was recruited along with actin to EA invasion sites with colocalization of both proteins (Figure 1C). Taken together, these first results showed that recruitment of Cdc42, Rac1 and RhoA GTPases by EAs is independent of their canonical actin-rich structures.

### Depletion of Rac1 but not RhoA inhibits EA internalization

**Figure 2:**
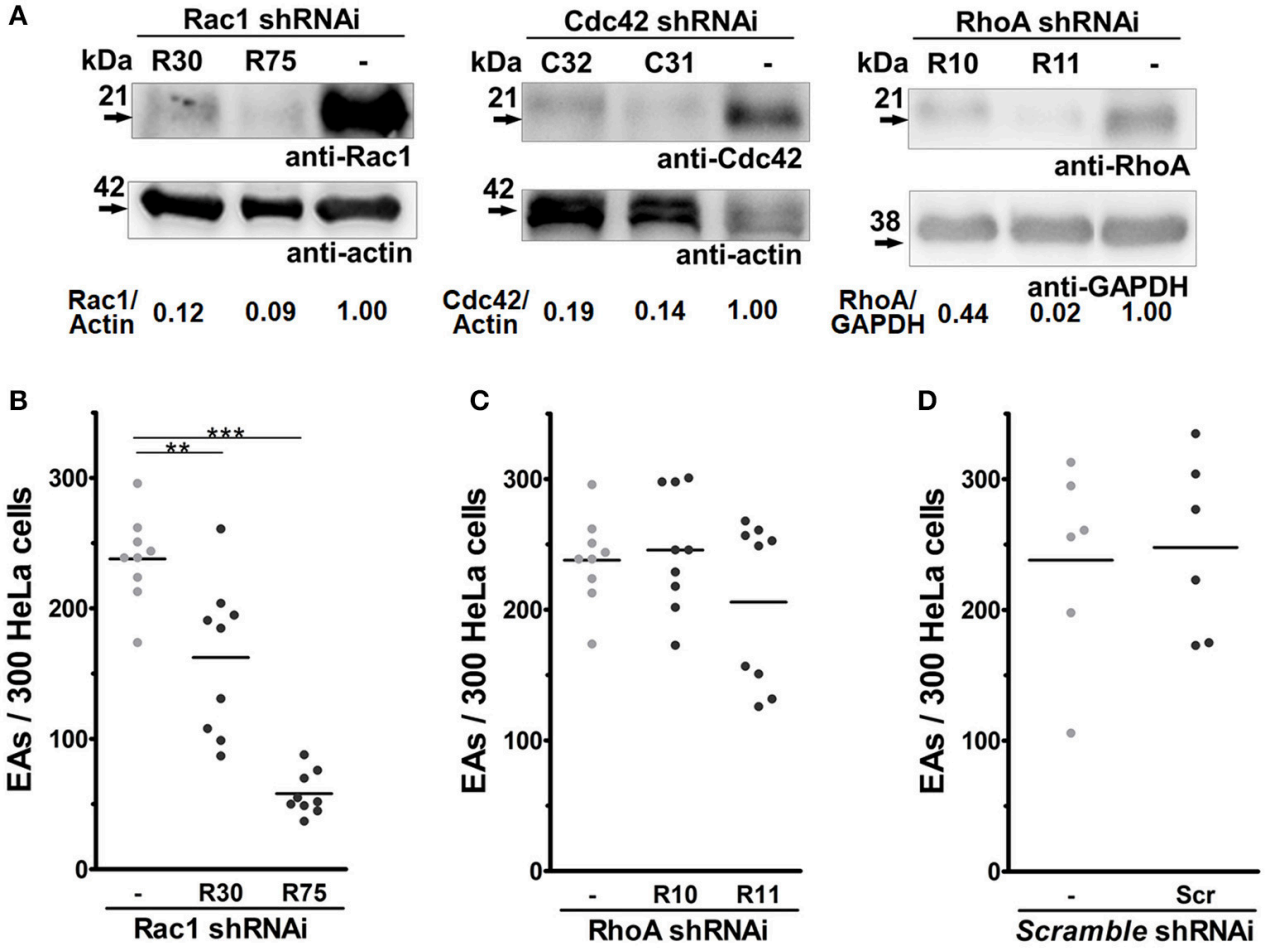
Depletion of Rac1, but not RhoA, reduces EA internalization by host cells. HeLa cells depleted of Rho-family GTPases were incubated with EA for 2 h, fixed with Bouin, and stained with Giemsa, and internalized parasites were quantified by optical microscopy. **(A)** Lysates of HeLa cells transduced by lentivirus containing shRNAi sequences and treated with puromycin for 2 weeks were evaluated for Rho GTPases expression by western blotting using actin or GAPDH as loading controls (detailed in Materials and Methods). **(B)** EA internalization was reduced approximately 35 and 76% in Rac1 depleted cell lineages (R30 and R75, respectively) when compared to the non-transduced group (-). **(C)** Cells depleted for RhoA had similar EA internalization when compared to the non-transduced groups (-). **(D)** EA invasion in cells transduced with scramble RNA sequences displayed no difference when compared to a non-transduced group (-). Results represent mean of at least three independent experiments in triplicate, and each dot represents one replicate (One-Way ANOVA, ^***^P < 0.001, ^**^P < 0.01, *n* ≥ 9).

Rac1, Cdc42, and RhoA display different roles during the invasion of intracellular pathogens (Lemichez and Aktories, 2013). Thus, even though no differences were observed in their recruitment by EAs, we generated stably depleted HeLa cells by lentiviral transduction, to answer whether these proteins regulate EA internalization (Bonfim-Melo et al., 2015). Two shRNAi sequences were used for each target protein and depletion was successfully obtained for Rac1 (R30 and R75 sequences), Cdc42 (C31 and C32 sequences) and RhoA (R10 and R11 sequences) as shown in Figure 2A. EA invasion was reduced in both Rac1-tranduced cell lines when compared to the non-transduced control group (-), reaching a mean of 76% inhibition in the cell line with smallest protein expression (R75), which suggests that Rac1 plays a positive role in EA host cell invasion (Figure 2B). In cells depleted of RhoA, no differences were observed between the depleted lineages (R10 and R11) and the non-transduced group (Figure 2C). Finally, there were also no differences in EA invasion between non-transduced (-) and scramble shRNAi transduced sequences (Scr), showing that EA invasion was not affected by the transduction procedure (Figure 2D). For cells depleted of Cdc42, a number of experiments (*n* ≥ 7) resulted in reduced internalization (Figure S1A), and a group of experiments (*n* ≥ 7) showed no significant differences between the depleted and control groups (Figure S1B), thus precluding a definite conclusion for this GTPase. Interaction of EAs with these depleted cells was also evaluated under scanning electron microscopy revealing no differences in mobilization or morphology of HeLa surface microvilli induced by the parasites (Figure S3, upper and central panels). Altogether, these results suggest that Rac1, but not RhoA, plays a positive role during EA internalization in HeLa cells.

### EA internalization kinetics, but not actin recruitment, is disturbed in Rac1 depleted cells

**Figure 3:**
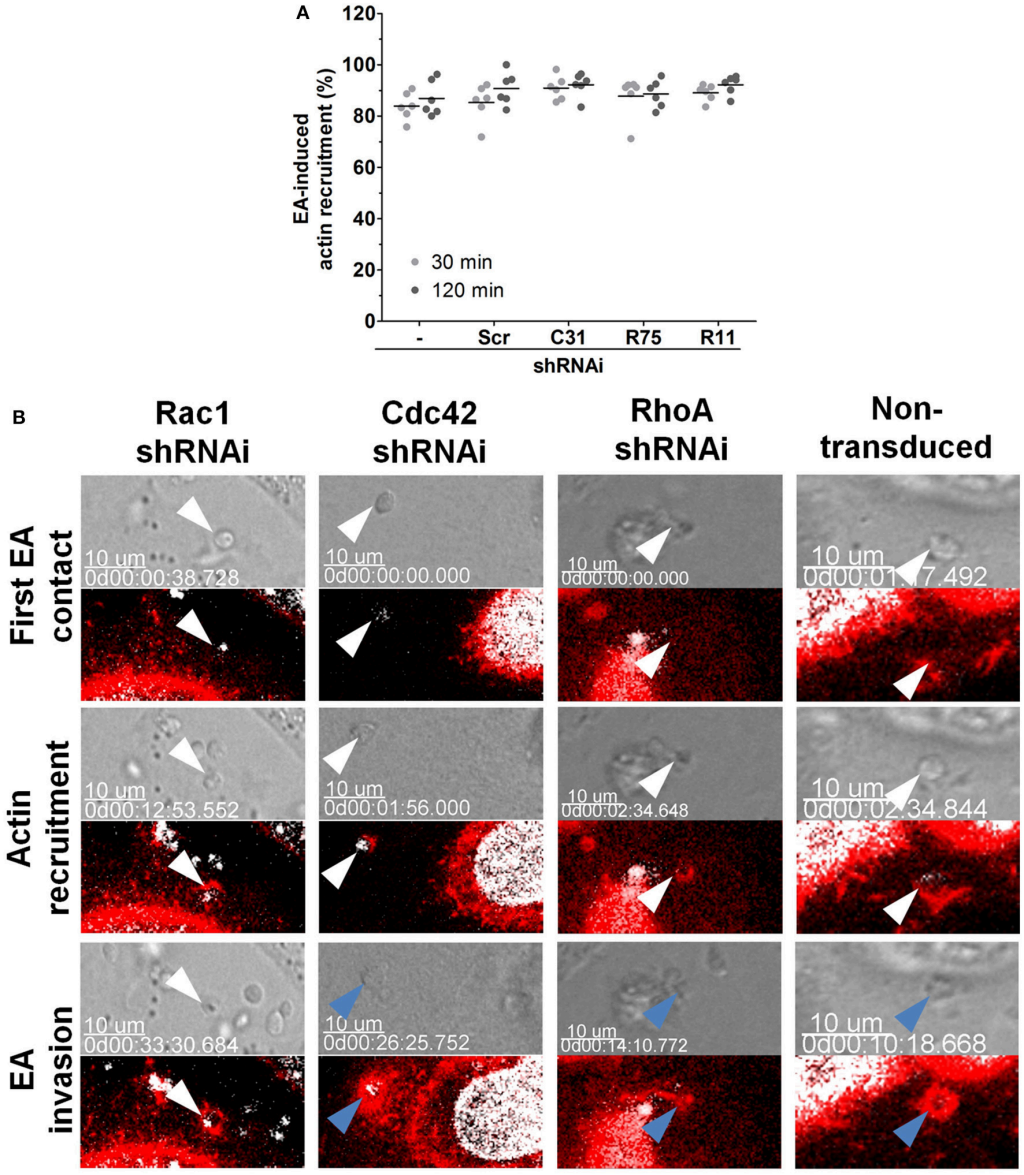
Depletion of Rac1 delays EA internalization. **(A)** Depleted HeLa lineages incubated with EA for the indicated times points were fixed and stained for filamentous actin, and the proportion of EA-induced actin recruitment was quantified by epifluorescence microscopy. Approximately 80% of interacting EAs induced actin recruitment in cells depleted of Cdc42, Rac1 or RhoA (C31, R75 or R11, respectively) and in the control [scramble (Scr) and non-transduced (-)] cell lineages at 30 or 120 min of interaction. Results represent mean of two independent experiments in triplicate, and each dot represents a replicate (One-Way ANOVA, no statistical differences, *n* ≥ 6). **(B)** EA-induced actin recruitment and internalization were assessed in the indicated depleted lineages by live time-lapse confocal microscopy. In Rac1 depleted cells (left panel), EA (white arrowheads) attaches and induces actin polymerization but fails to invade even after 30 min of initial contact. In contrast, in the non-transduced control group (right panel) and in the Cdc42 or RhoA depleted HeLa cells (central panels), EAs (white arrowheads) attach, induce actin (red) polymerization and successfully invade (blue arrowhead) host cells a few minutes after their first contact. LifeAct-RFP (red) plasmid transfection was used to track filamentous actin, and Hoechst (white) was used to stain nuclei and kinetoplasts. Corresponding DIC images of single focal planes and merged fluorescence channels were acquired with approximately 2-min time intervals. These are representative observations from at least 50 interactions of 2 independent experiments.

Rho GTPases perform their roles mainly by modulating microfilament rearrangements (Spiering and Hodgson, 2011). To evaluate whether distinct EA invasion phenotypes in depleted HeLa cells were due to actin rearrangements, EAs were incubated with these cells for 30 or 120 min and the proportion of EA-induced actin recruitments were assessed under epifluorescence microscopy. No statistically significant differences in the proportion of actin recruitments were observed between the depleted (R75, C31, and R11) and control (non-transduced and scramble) groups (Figure 3A). In addition to the proportion of actin recruitments, actin staining intensity at EA invasion sites was evaluated. Only cells depleted of Rac1 were observed to have increased actin staining after 120 min of interaction in comparison to non-transduced control groups (Figure S2). Mobilization of HeLa cell surface microvilli by EA attachment was also not affected by Rho-family GTPase depletion as shown by scanning electron microscopy (Figure S3, top and central panels).

Actin restructuring is a very dynamic process, and its modulation can rely on cycles of polymerization/depolymerization and binding to partner proteins (Buchsbaum, 2007). Considering that the depletion of Rho-family GTPases in the majority of groups did not cause notable changes in actin polymerization at EA invasion sites, we evaluated actin recruitment during EA internalization in live cells by time-lapse experiments under confocal microscopy. In non-transduced cells, EAs (white arrowheads) attached to the host cell surface, induced actin recruitment and were engulfed after few minutes of initial contact (Figure 3B, right panel, Video 3), corroborating our previous observations that EAs can be internalized by HeLa cells only a few minutes after their initial contact (Fernandes et al., 2013; Bonfim-Melo et al., 2015). A similar entry pattern was observed in cells depleted of Cdc42 and RhoA (Figure 3B, central panels, Videos 4, 5) and in the scramble transduced group (Video 7). However, in cells depleted of Rac1, EAs (white arrowheads) attached and promoted actin recruitment but failed to be internalized by HeLa cells when similar time periods were compared to the non-transduced control groups (Figure 3, left panel, Video 6). Even 33 min after first contact, the indicated EA (white arrowhead) did not invade the host cell. Together, these experiments suggest that inhibition of EA invasion in cells depleted of Rac1 could be related to delayed EA internalization but not to a reduction in polymerized actin.

### Rac1 and RhoA GTPase activation alters EA-induced actin recruitment in host cells

**Figure 4:**
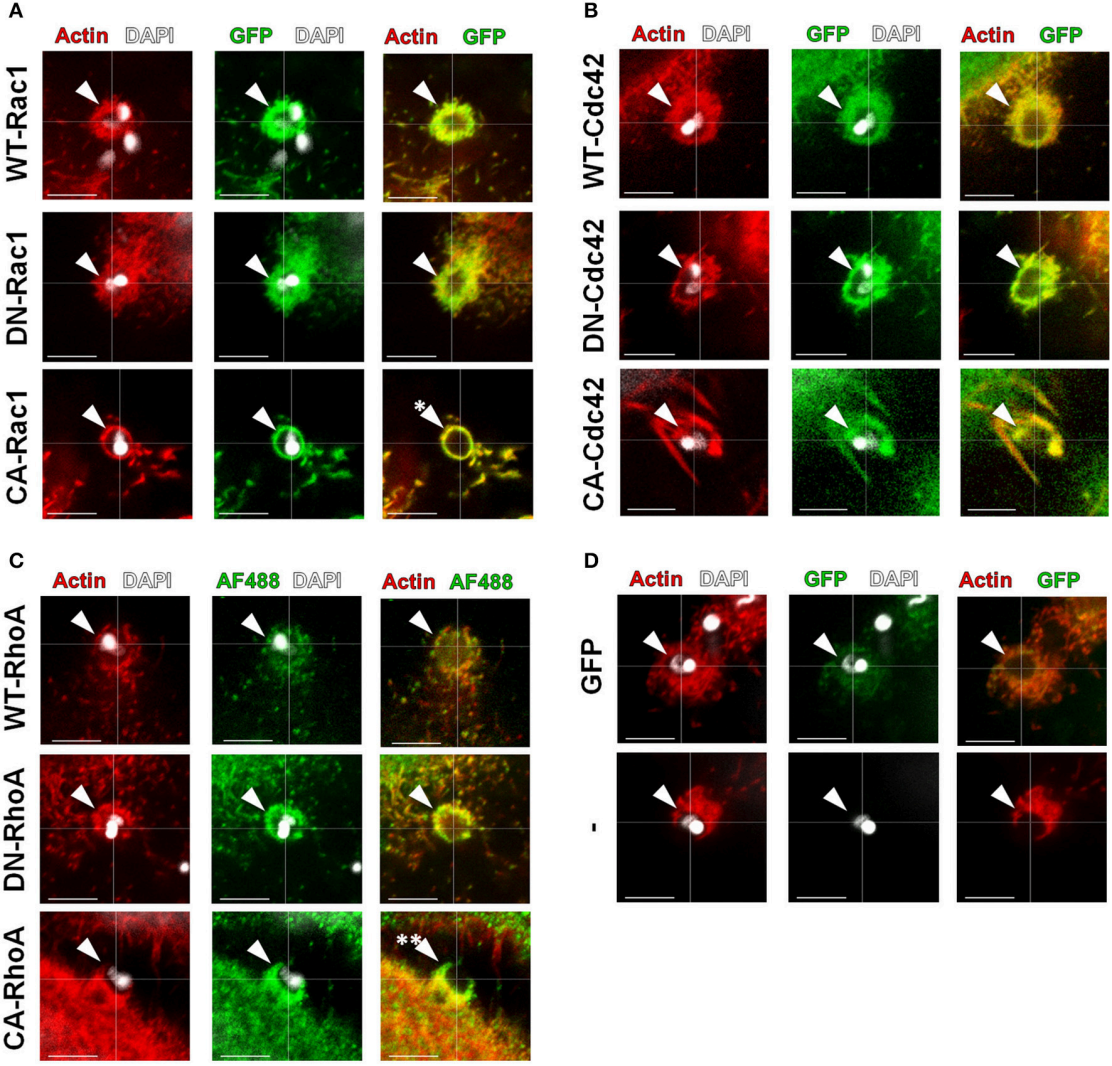
Expression of activation constructs of Rac1 and RhoA altered the morphology of EA-induced actin recruitment. HeLa cells were transfected with WT, DN or CA mutated GTPases (green) and incubated with EAs for 1 h, and their recruitment (arrowheads) along with actin (red) was evaluated by confocal microscopy. **(A)** Recruitment and colocalization with actin to EA invasion sites of the indicated Rac1 constructs with morphological alterations only in the CA-Rac1 transfected group (single asterisk) compared to the DN and WT control groups. **(B)** Recruitment and colocalization with actin of the Cdc42 constructs (GFP tagged; green) without drastic morphology alterations when comparing the DN and CA groups to the WT control group. **(C)** Recruitment and colocalization with actin of WT and DN RhoA constructs (His-tagged, Alexa Flour 488 secondary; green). CA-RhoA expression induced discrete actin recruitment, leading to its partial colocalization with actin (double asterisk). **(D)** No alteration in EA-induced actin-rich cup-like structures in cells transfected with cytosolic GFP alone (GFP) or in non-transfected cells (−). Single focal plane images with phalloidin-TRITC (red) staining filamentous actin and DAPI (white) staining EA nuclei and kinetoplasts. These are representative observations from at least 50 interactions of 2 independent experiments. Bars = 5 μm.

GTP-bound GTPases bind to and activate effector proteins, inducing actin rearrangements, in a variety of cellular processes (Spiering and Hodgson, 2011). We transfected HeLa cells with GTP or GDP bound mutants (constitutively active (CA) or dominant negative (DN), respectively) and observed their recruitment to EA invasion sites by confocal microscopy. Although both DN and CA constructs were similarly recruited to EA invasion sites (arrowheads) when compared to the native isoform (WT), we observed that actin and Rac1 recruiting projections were restructured as thin lamellae in CA groups (Figure 4A, single asterisk). Similar structures were not exclusive to EA cups; they were also found in other regions of the cell body of CA-Rac1 transfected cells, suggesting that Rac1 is effectively active in overexpressing cells as previously described (Hall, 1998). In cells transfected with Cdc42 isoforms, no noticeable morphological differences in actin and Cdc42 recruitments (arrowheads) were observed between DN and CA isoforms when compared to the wild type (WT) protein (Figure 4B). On the other hand, for cells expressing CA-RhoA, reduced actin staining at EA invasion sites when compared to the WT or DN groups was detected. In the merged panel, it is possible to distinguish a region of CA-RhoA (green) labeling without colocalization with filamentous actin (red) (Figure 4C, double asterisk). EA interactions were also evaluated in cells expressing cytosolic GFP alone or non-transfected ones (-) as controls of non-specific GFP recruitment and changes in actin morphology due to the transfection protocol, respectively (Figure 4D).

### Rho-family GTPase activation disparately alters EA internalization by host cells

**Figure 5:**
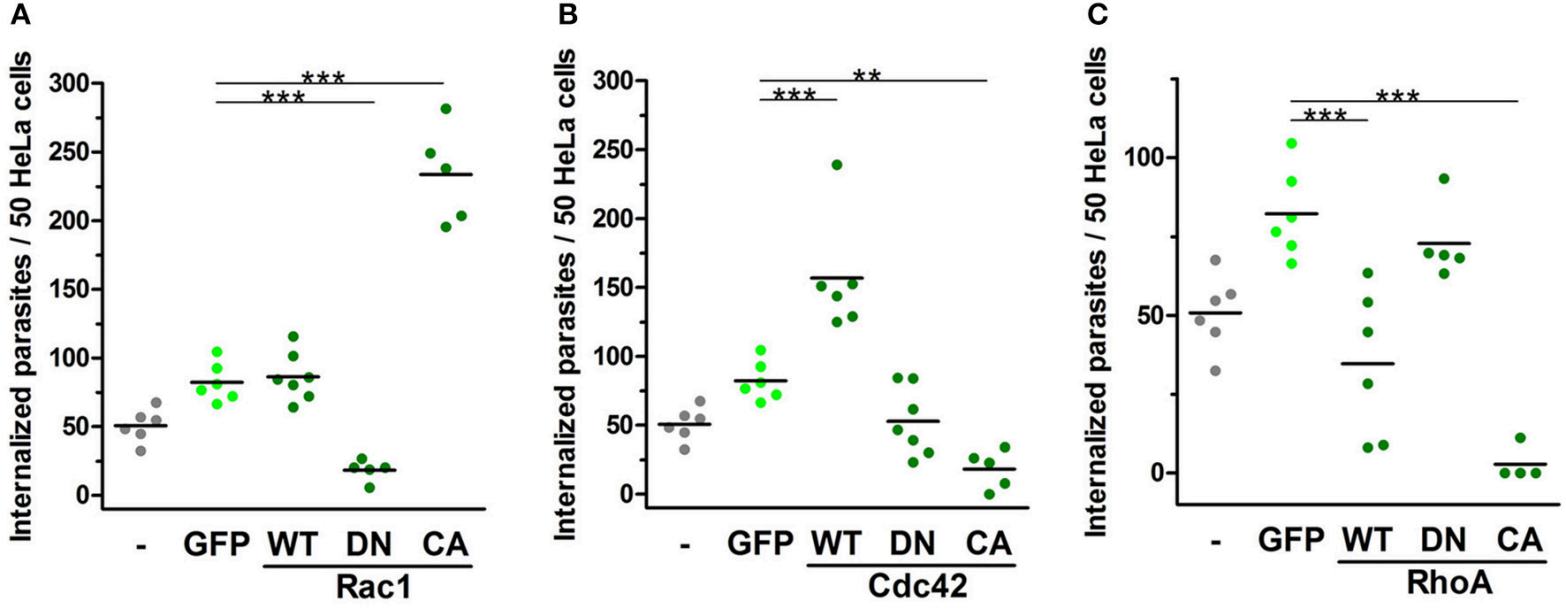
EA internalization is altered by the expression of activation mutated Rho-family GTPases. HeLa cells transfected with WT, DN or CA mutated GTPases were incubated with EAs (MOI 10) for 2 h, and intracellular parasites were quantified under epifluorescence microscope in transfected cells. Cells transfected with cytosolic GFP (GFP) were used as a control group and displayed similar EA internalization as non-transfected cells (-). **(A)** Cells expressing CA-Rac1 displayed increased EA internalization, and cells expressing DN-Rac1 displayed reduced EA internalization compared to the GFP control group. **(B)** For Cdc42, cells expressing WT construct had increased EA internalization and cells expressing CA construct had reduced EA internalization compared to the GFP control group. **(C)** Cells transfected with WT-RhoA or CA displayed reduced EA internalization compared to the GFP control group. Results represent mean of two independent experiments in triplicate or duplicate, and each dot represents one replicate (One-Way ANOVA, ^***^P < 0.001, ^**^P < 0.01, *n* ≥ 4).

In 2004, our group showed the positive role of Rac1 during the invasion of G strain EAs in MDCK cell lines overexpressing mutated Rho-family GTPases (Fernandes and Mortara, 2004). Herein, we evaluated the role of Rho-family GTPases activation using HeLa cells transfected with comparable constructs. Cells transfected with cytosolic GFP were similarly invaded by EA compared to non-transfected cells and were used as controls for these experiments. EA internalization in cells expressing native constructs of Rac1 was similar to that of the GFP control group (Figure 5A and Figure S4A). In contrast, EA invasion was reduced in cells expressing DN-Rac1 and accordingly increased in cells expressing CA-Rac1 (Figure 5A and Figure S4A). For cells expressing Cdc42 native protein, we observed increased EA internalization; however, surprisingly, in cells expressing CA-Cdc42, we had reduced internalization of the parasite (Figure 5B and Figure S4A). This particular observation suggests that cycling between active and inactive states is important for Cdc42 participation in EA host cell invasion rather than its activation alone, which is similar to what is observed in phagocytosis (Beemiller et al., 2010). No differences were observed in DN-Cdc42 when compared to the GFP control group (Figure 5B and Figure S4A). Finally, EA invasion was reduced in cells expressing WT-RhoA or CA-RhoA but unaltered in cells expressing the DN isoform (Figure 5C and Figure S4A). In cells expressing CA-RhoA, stress fibers (white arrowheads, Figure S4B) were denser and more numerous compared to non-transfected cells in the same group (blue arrowheads, Figure S4B) and to the group of non-transfected cells (Figure S4A). Together, these results show that Rho-family GTPase activation specifically modulates EA internalization in HeLa cells.

### Rho GTPase effector proteins, WAVE-2 and N-WASP, participate in EA internalization

**Figure 6:**
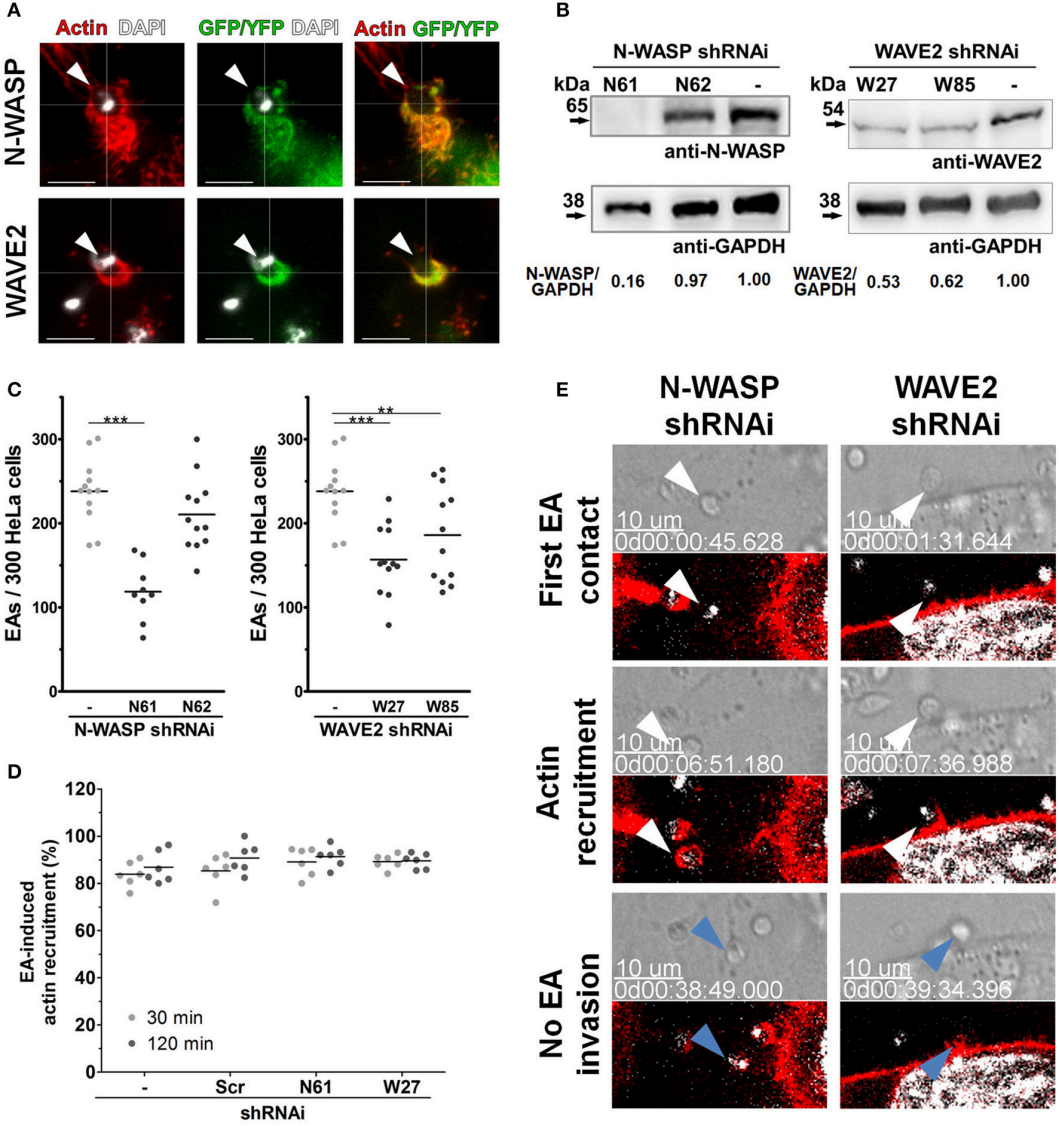
Rho-family GTPase effector proteins, N-WASP and WAVE2, participate in EA invasion of HeLa cells. **(A)** HeLa cells were transfected with N-WASP-GFP or WAVE2-YFP and incubated with EAs for 1 h and interactions were evaluated by confocal microscopy. Both N-WASP (green) and WAVE2 (green) are recruited and colocalize with actin (red) at EA invasion sites (arrowheads). **(B)** Lysates of HeLa cells transduced by lentivirus containing shRNAi sequences and treated with puromycin for 2 weeks were evaluated for N-WASP and WAVE2 expression by western blotting using GAPDH as loading controls (detailed in Materials and Methods). **(C)** EA internalization was reduced in cells transduced with the N-WASP shRNAi N61 sequence (with confirmed N-WASP depletion, **B**) but not with the N62 sequence (no N-WASP depletion, **B**). EA invasion was also reduced in cells depleted of WAVE2 (depletion in both transduced sequences, W27 and W85, **C**) when compared to the non-transduced control group. Results represent mean of at least three independent experiments in triplicate, and each dot represents one replicate (One-Way ANOVA, ^***^P < 0.001, ^**^P < 0.01, *n* ≥ 9). **(D)** Depleted HeLa lineages incubated with EA for the indicated times points were fixed and stained for filamentous actin, and the proportion of EA-induced actin recruitment was quantified in an epifluorescence microscope. Approximately 80% of interacting EAs induced actin recruitment in cells depleted of N-WASP or WAVE2 (N61 or W27, respectively) and in the control [scramble (Scr) and non-transduced (-)] cell lineages at 30 or 120 min of interaction. Results represent mean of two independent experiments in triplicate, and each dot represents a replicate (One-Way ANOVA, no statistical differences, *n* ≥ 6). **(E)** HeLa cells depleted of N-WASP (N61, left panel) or WAVE2 (W27, right panel) were transfected with LifeAct-RFP (red) and incubated with EA, and interactions were evaluated by time-lapse confocal microscopy experiments. In both cell lineages, EAs (white arrowheads) attach and induce actin polymerization but fail (blue arrows) to invade after similar interaction times compared to the non-transduced control group (see right panel of Figure 3). Hoechst (white) was used to stain nuclei and kinetoplasts. DIC and single focal plane of merged fluorescence channels were acquired with a 45 s interval. These are representative observations from at least 50 interactions of two independent experiments.

Rac1 and Cdc42 activity is mainly mediated by their effector proteins, WAVE-2 and N-WASP, respectively, which promote actin polymerization after binding to and activating the Arp2/3 complex (Spiering and Hodgson, 2011). Using similar approaches to those used for Rho-family GTPases, we evaluated WAVE2 and N-WASP involvement in the actin-dependent mechanism of EA host cell invasion. In cells transfected with fluorescently labeled N-WASP or WAVE2, we first observed that both proteins are recruited to and colocalize with actin at EA invasion sites (arrowheads, Figure 6A). EA invasion reduction was approximately 51% in the N61 cell lineage (N-WASP depleted) and 34% in the W27 cell lineage (WAVE2 depleted) (Figure 6C). Despite this reduction, inhibition of EA invasion was not followed by mobilization or morphology changes of HeLa surface microvilli (Figure S3, bottom panels) nor by changes in the proportion of actin recruitments to EA invasion sites (Figure 6D), which was similar to Rac1 depleted cell observations. However, time-lapse experiments revealed that EAs induced actin polymerization but failed to invade (Figure 6E, Videos 8, 9) these cells during similar periods evaluated in parallel control groups (Figure 3, right panel, blue arrowheads). Altogether, these results show that N-WASP and WAVE2 also participate in EA host cell invasion, possibly as partner molecules of Cdc42 and Rac1 GTPases, respectively.

## Discussion

EAs are able to sustain the *T. cruzi* life cycle in mammals (Ley et al., 1988; Lima et al., 2010), and since the first reports, participation of the host cell actin cytoskeleton and other partner molecules of the membrane and extracellular matrix were shown as crucial participants in EA uptake by host cells (Mortara, 1991; Procópio et al., 1999). Recently, our group described EA induction of coordinated recruitment of PIP_2_ and PIP_3_ markers during the invasion of host cells, corroborating the hypothesis of a phagocytosis-like mechanism of EA entry in non-phagocytic HeLa cells (Fernandes et al., 2013). Herein we were able to elucidate aspects that were not previously assessed regarding host cell Rho-GTPases and EAs invasion (Fernandes and Mortara, 2004). Trypomastigote mechanisms of invasion rely mostly on host cell lysosome exocytosis and actin participation remains controversial (de Souza et al., 2010) so it would be inappropriate to compare our findings with those obtained with trypomastigotes (Fernandes and Mortara, 2004; Dutra et al., 2005). Considering the observations from our previous work, the present study aimed to evaluate host cell Rho-family GTPases and their effector proteins in actin dynamics during HeLa cell invasion by EAs, possibly providing new insights into this recently proposed mechanism for parasite entry.

Rac1 and WAVE2 are both activated and recruited to the plasma membrane after binding to PIP_3_, where they can promote actin polymerization (Takenawa and Suetsugu, 2007; Spiering and Hodgson, 2011). Rac1 recruitment observed herein could be a response to PIP_3_ formation, which is present in phagocytosis-like processes, including the one described for EA host cell invasion (Fernandes et al., 2013; Campa et al., 2015). Rac1 may also be a downstream effector of the host PI3k-dependent pathway, which was previously described by our group as being another pathway of possible involvement of Rac1 during EA internalization (Fernandes et al., 2006; Campa et al., 2015). Stimulation of the well-described EGFR and Vav2 axis (Buchsbaum, 2007; Patel et al., 2007) can be a signaling pathway that possibly leads to Rac1 activation as interactions of EAs with host cells stimulate the corresponding receptor (Bonfim-Melo, unpublished). Invasion assays using depleted or overexpressing cells showed that Rac1 interfered with EA invasion, although these studies were conducted without inhibiting the amount of polymerized actin at EA invasion sites as shown by evaluated recruitment assays with fixed samples (Figure 3). Even though Rac1 modulates actin polymerization, there are other proteins with related activities that could compensate for Rac1 absence/inactivation, including Cdc42, which could explain why a reduction in actin recruitment in these cells was not observed (Pollard and Cooper, 2009; Spiering and Hodgson, 2011). In fact, an increase in actin accumulation at EA interacting sites in Rac1 depleted cells after 120 min of incubation may suggest that despite no internalization, actin polymerization occurred (Figure S2). Together, these results may suggest that Rac1, rather than promoting actin polymerization in an independent manner, can activate and interact with key microfilament signaling proteins, such as cortactin, dynamin2 and PAK1/2 (Weed et al., 1998; Barrias et al., 2010), which are mainly important in the later stages of EA internalization. Nevertheless, the results obtained here showed that Rac1 is a key regulator of EA internalization.

We observed that RhoA activation reduced EA internalization in HeLa cells overexpressing the CA isoform (Figure 5C), although no differences were observed in invasion assays using RhoA depleted cells (Figure 2D). RhoA is an important GTPase, rearranging actin filaments and driving the formation of stress fibers, and in our work, we observed that overexpression of its CA mutant isoform led to denser and more numerous stress fibers, which is similar to previous reports (Nobes and Hall, 1995; Hall, 1998). This signaling redirection probably led to the inhibition in actin recruitment observed at EA invasion sites (Figure 4C), and it is probably the main mechanism of the negative effect of RhoA on EA internalization (Figure 5C). Since the positive role of Rac1 in EA internalization was demonstrated, negative regulation by RhoA can also be an alternative/additional pathway of inhibition of actin dynamics at EA invasion sites (Guilluy et al., 2011). Mechanisms reporting RhoA activity and the consequent inhibition of Rac1 activity have been previously described and involve proteins such as RhoA effector ROCK and Rac1 upstream GAP, such as Fil-GAP and ArhGAP22 (Ohta et al., 2006; Sanz-Moreno et al., 2008). Overexpressing mutant constructs and confocal microscopy experiments revealed an intricate Rho GTPase control of actin dynamics during EA internalization in HeLa cells, which was more complex than what we previously described for MDCK cells (Fernandes and Mortara, 2004). Different effects were also seen in HeLa and Vero cells depleted of Rho GTPases or WASP proteins in *Listeria* ssp. invasion assays, showing that other pathogens can also differently regulate these pathways in a cell-dependent manner (Bierne et al., 2005). Thus, our work suggests Rac1 as positive and RhoA as negative regulators of EA internalization in HeLa cells.

Regarding Cdc42, it can bind to and activate N-WASP, promoting actin polymerization by the Arp2/3 complex and participating in a range of actin-related cellular processes (Spiering and Hodgson, 2011). Cdc42 can also activate mDia2 to promote actin polymerization independently of the Arp2/3-N-WASP pathway and regulate severing of actin filaments through the PAK1-LIMK-cofilin pathway, showing possible actin-related mechanisms controlled by this Rho GTPase (Edwards et al., 1999; Lammers et al., 2008). However, our invasion and actin recruitment results using HeLa cells depleted of Cdc42 suggested that this GTPase role in actin dynamic regulation during EA internalization can at least partially be compensated by other proteins. Not only Cdc42 but also Rac1 can be activated by Vav2 downstream of EGFR, representing one possible mechanism to compensate for Cdc42 depletion (Buchsbaum, 2007). Cdc42 participation also fits our hypothesis of EA-induced phagocytosis, as Cdc42 can be recruited and activated by PIP_2_ at initial interaction time points (Levin et al., 2015). Additionally, reduced EA internalization in cells overexpressing the CA-Cdc42 isoform was also shown to occur during phagocytosis in which its permanent activation inhibits actin depolymerization needed for phagocytic cup sealing (Beemiller et al., 2010). However, considering the phagocytosis mechanism, CA-Cdc42 is able to induce constant PI3k activation and PIP_3_ production, which can promote the recruitment of Rac1 GAPs and possibly inhibit EA internalization by a Rac1-inhibitory pathway (Campa et al., 2015). The fact that no alterations (DN-Cdc42) or increases (WT-Cdc42) in EA internalization have been observed here shows that cycling between active and inactive states is important for Cdc42 participation in EA host cell invasion rather than its activation alone, as previously described in phagocytosis (Beemiller et al., 2010). Thus, our results corroborate Cdc42 participation in EA uptake; however, it can possibly be compensated by Rac1 to some extent and Cdc42 might also regulate Rac1 through the PI3k pathway involved in phagocytosis mechanisms.

Additionally, based on our previous work (Fernandes and Mortara, 2004), we also evaluated the participation of Rho-family GTPase effectors N-WASP and WAVE2 during EA invasion to study the partners and downstream signals of these protein family members that lead to actin polymerization. Our results confirmed the contribution of N-WASP and WAVE2, which could promote actin polymerization downstream of Cdc42 and Rac1, respectively (Padrick and Rosen, 2010). Similar to Cdc42, N-WASP can be activated by the PIP_2_ binding produced during EA internalization; however, other parallel mechanisms, such as phosphorylation, interactions with SH3 containing proteins (such as cortactin) or even oligomerization cannot be discarded (Padrick and Rosen, 2010; Fernandes et al., 2013; Bonfim-Melo et al., 2015). These alternative activation mechanisms may explain why we observed more evident perturbation of EA invasion in cells depleted for N-WASP when compared to cells depleted for Cdc42, even though they are both partners in the same Arp2/3 complex actin-dependent signaling pathway. Thus, N-WASP involvement might occur independently of the Cdc42 activation mechanism. On the other hand, WAVE-2 participation in EA uptake is likely to occur downstream of Rac1 since its activity is tightly regulated by this GTPase in addition to the scarce number of reports describing the Rac1-independent effector activity of WAVE2. WAVE2 may also cooperate with cortactin and PKD1 during EA internalization (Bonfim-Melo et al., 2015) as it does in lamellipodia protrusions (Eiseler et al., 2010). Additionally, residual EA invasion rates in WAVE2 depleted cells might occur due endogenous WAVE1 since this isoform can partially compensate for the absence of WAVE2 in actin polymerization as already described in other models (Bierne et al., 2005; Padrick and Rosen, 2010). Thus, regarding Rho GTPase effector proteins, N-WASP is possibly acting independently of Cdc42, whereas WAVE2 is possibly acting as a partner of Rac1 during EA internalization in HeLa cells.

**Figure 7:**
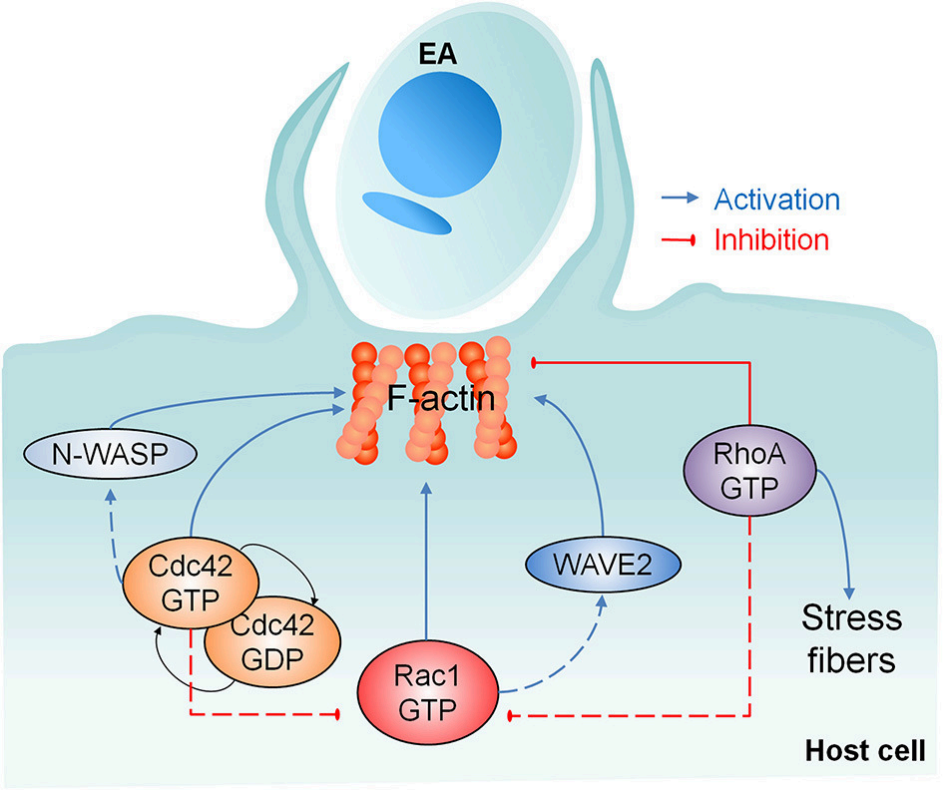
Involvement of Rho-family GTPases and their effector proteins in actin mediated EA host cell invasion. Our work showed the recruitment of Rho-family GTPases, N-WASP and WAVE2 after EA contact with the host cell. None of these proteins alone is required for F-actin polymerization, but Cdc42 cycling between active/inactive states and Rac1 activation are important for EA internalization. RhoA promotes actin polymerization in stress fibers but inhibits this process at EA invasion sites, which abolishes parasite entry and reveals the negative role of RhoA in this process. Dashed lines represent the possible joint participation of Rac1/WAVE2 and Cdc42/N-WASP and the possible negative regulation mechanisms of Cdc42 and RhoA over Rac1 activity.

Collectively, our results provide evidence of the positive participation of Cdc42, N-WASP, WAVE2 and most importantly Rac1 in actin-dependent EA host cell invasion, but with parallel and negative regulation by RhoA as shown in Figure 7. These findings also corroborate our previous work showing that EAs engage a phagocytosis-like mechanism when invading non-professional phagocytic cells (Fernandes et al., 2013). The identification of additional partner proteins of Rho-family GTPases as well as N-WASP and WAVE-2 will provide further insights into the molecular mechanisms controlling microfilament rearrangements in host cell invasion by EAs.

## Author contribution

AB-M and RM conceived and acquired the funding for the project. AB-M and ÉF performed experiments, formatted datasets, and analyzed data. AB-M, ÉF, and RM wrote the manuscript interpreted data and contributed to the intellectual content of this work.

### Conflict of interest statement

The authors declare that the research was conducted in the absence of any commercial or financial relationships that could be construed as a potential conflict of interest.
