# Supplementary figures and images for: Rac1/WAVE2 and Cdc42/N-WASP Participation in Actin-Dependent Host Cell Invasion by Extracellular Amastigotes of Trypanosoma cruzi
Source: Front Microbiol. 2018 Feb 28;9:360. doi: 10.3389/fmicb.2018.00360 (PMC5835522; doi:10.3389/fmicb.2018.00360)

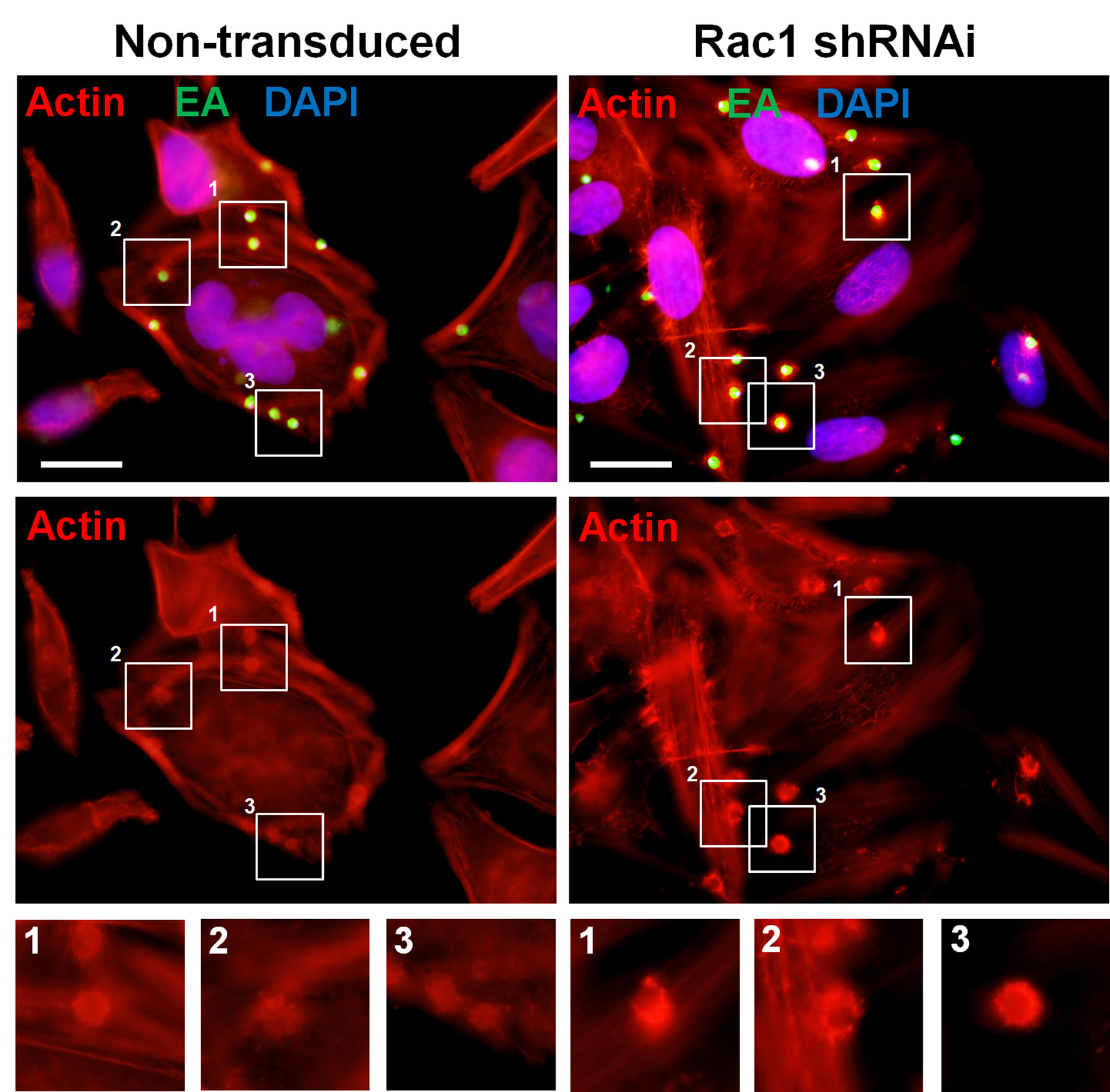

Supplement: Supplementary file 11 [file Image2.TIF]

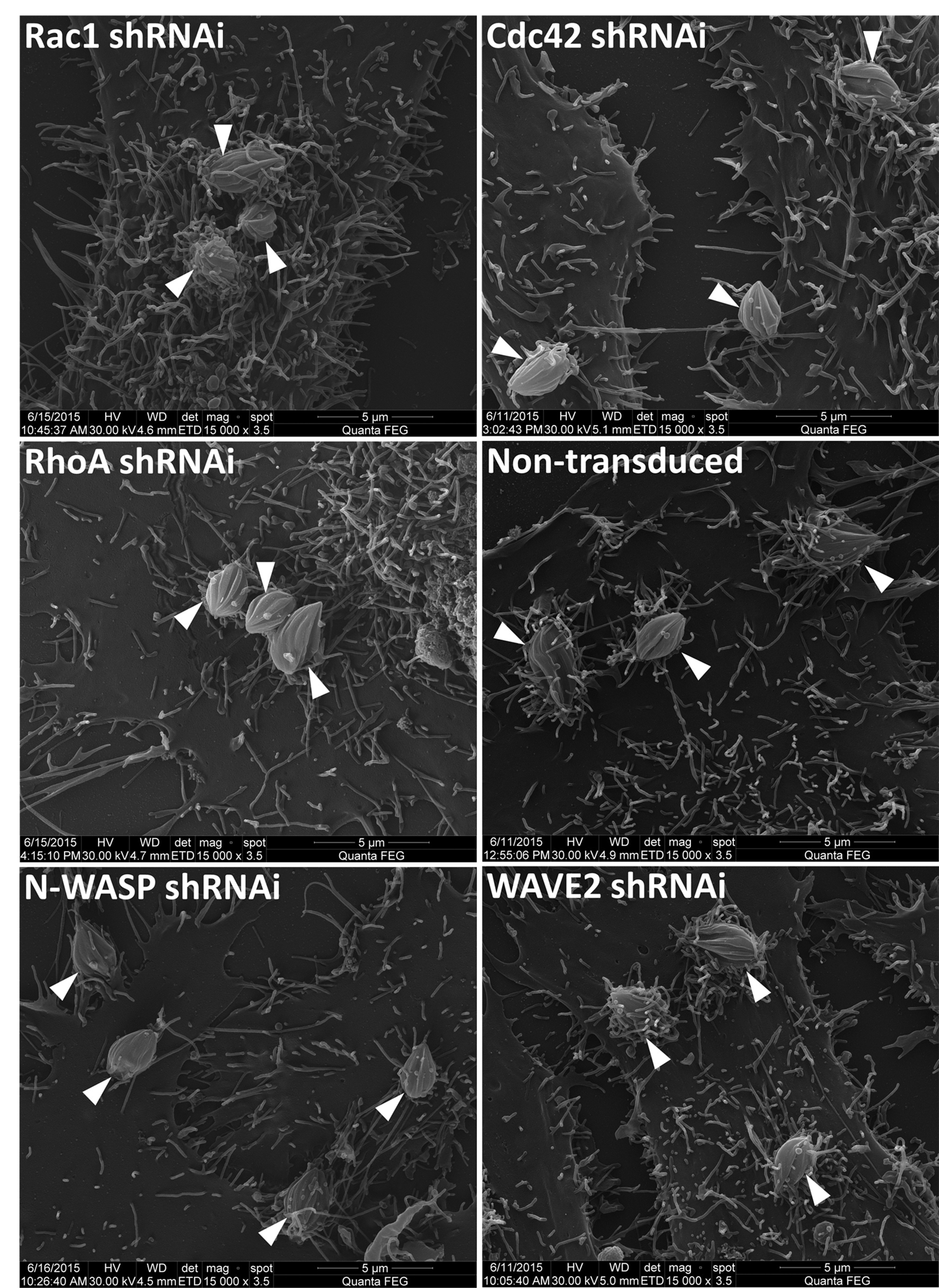

Supplement: Supplementary file 12 [file Image3.TIF]

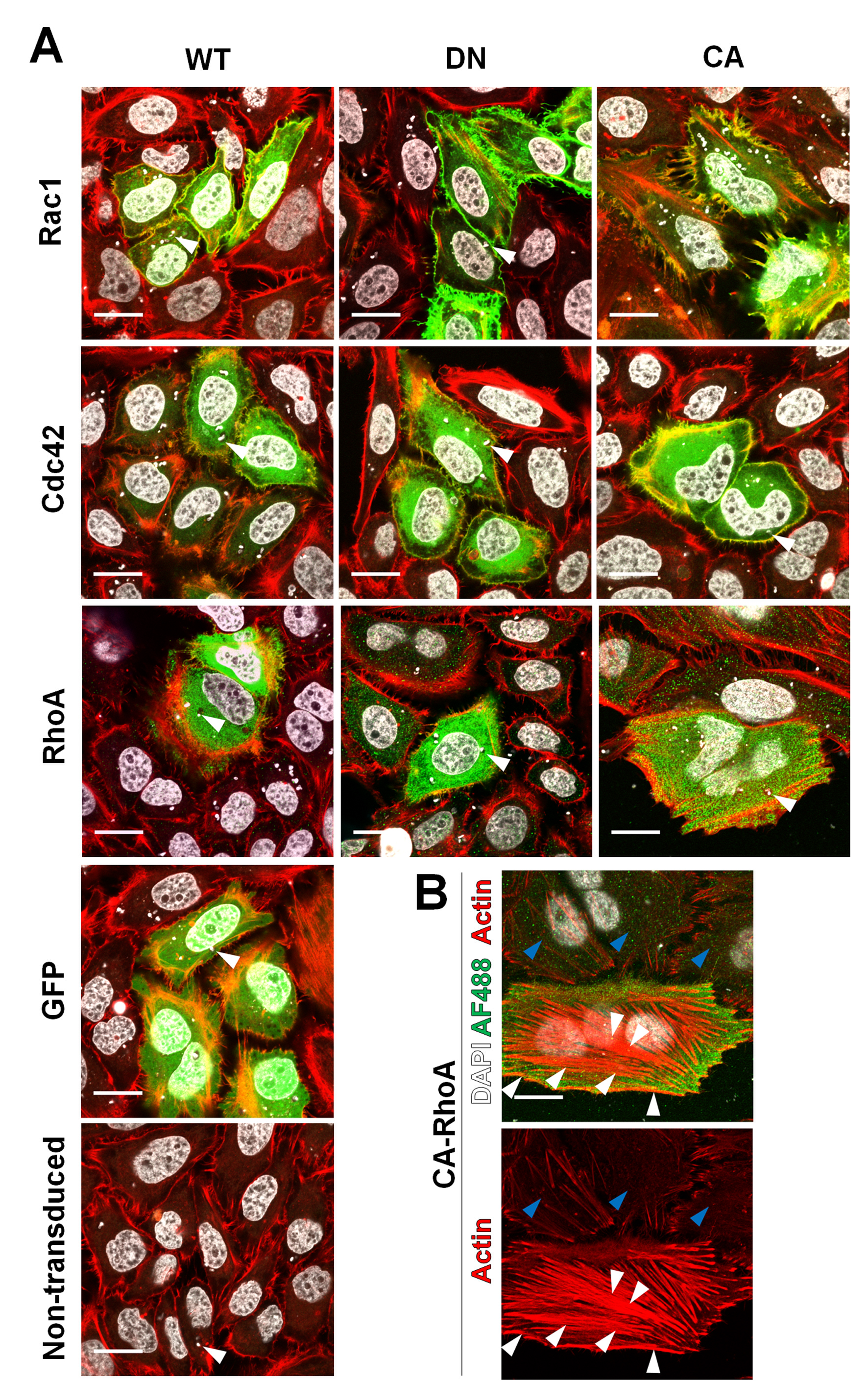

Supplement: Supplementary file 13 [file Image4.TIF]
