# Supplementary material for: Rac1/WAVE2 and Cdc42/N-WASP Participation in Actin-Dependent Host Cell Invasion by Extracellular Amastigotes of Trypanosoma cruzi
Source: Front Microbiol. 2018 Feb 28;9:360. doi: 10.3389/fmicb.2018.00360 (PMC5835522; doi:10.3389/fmicb.2018.00360)
Supplement: Supplementary file 14 [file DataSheet1.DOCX]

Supplementary Material

**Rac1/WAVE2 and Cdc42/ N-WASP participation in** **actin-dependent host cell invasion by Extracellular Amastigotes of *Trypanosoma cruzi***

**Alexis Bonfim-Melo^1, #^, Éden Ramalho Ferreira^1, #^ and Renato Arruda Mortara^1^**

*** Correspondence:** Corresponding Author: [ramortara@unifesp.br](mailto:ramortara@unifesp.br)

# Supplementary Data

**1.2 VIDEO LEGENDS**

**Video 1.** Host cell GTPases Rac1 and actin are recruited and colocalize during EA cellular invasion. HeLa cells were cotransfected with Rac1 (GFP tagged) and LifeAct (RFP tagged), and interactions with EAs were evaluated by live confocal microscopy. An EA (arrowheads) attaches and induces the formation of the cup-like structure rich in Rac1 and actin in HeLa cells, which pulls parasites inside the cell. Internalization is identified by the complete closure of the cup-like structure into a circle and the reduction in parasite refringence observed in DIC. The EA nucleus and kinetoplast were stained with Hoechst.

**Video 2.** Host cell GTPases Cdc42 and actin are recruited and colocalize during EA cellular invasion. HeLa cells were cotransfected with Cdc42 (GFP tagged) and LifeAct (RFP tagged), and interactions with EAs were evaluated by live confocal microscopy. An EA (arrowheads) attaches and induces the formation of the cup-like structure rich in Cdc42 and actin in HeLa cells, which pulls parasites inside the cell. Internalization is identified by the complete closure of the cup-like structure into a circle and the reduction in parasite refringence observed in DIC. The EA nucleus and kinetoplast were stained with Hoechst.

**Video 3.** Depletion of Rac1 delays EA internalization. EA-induced actin recruitment and internalization were assessed in the indicated depleted cell lines by live time-lapse confocal microscopy. In Rac1 depleted cells, EA (arrowheads) attaches and induces actin polymerization but fails to invade these cells after interaction times similar to the control groups (between 30 or 40 minutes). LifeAct-RFP (red) plasmid transfection was used to track filamentous actin, and the EA nucleus and kinetoplast were stained with Hoechst (white). These results are representative observations from at least 50 interactions of 2 independent experiments.

**Video 4.** Depletion of Cdc42 does not interfere with EA-induced actin recruitment and internalization. EA-induced actin recruitment and internalization were assessed in the indicated depleted cell lines by live time-lapse confocal microscopy. In Cdc42 depleted cells, EA (arrowheads) attaches, induces actin polymerization and invades (at 24 minutes frame) these cells after interaction times similar to the control groups (between 30 or 40 minutes). LifeAct-RFP (red) plasmid transfection was used to track filamentous actin, and the EA nucleus and kinetoplast were stained with Hoechst (white). These results are representative observations from at least 50 interactions of 2 independent experiments.

**Video 5.** Depletion of RhoA does not interfere with EA-induced actin recruitment and internalization. EA-induced actin recruitment and internalization were assessed in the indicated depleted cell lines by live time-lapse confocal microscopy. In Cdc42 depleted cells, EA (arrowheads) attaches, induces actin polymerization and invades (at 10 minutes frame) these cells after interaction times similar to the control groups (between 30 or 40 minutes). LifeAct-RFP (red) plasmid transfection was used to track filamentous actin, and the EA nucleus and kinetoplast were stained with Hoechst (white). These results are representative observations from at least 50 interactions of 2 independent experiments.

**Video 6.** EA-induced actin recruitment and internalization in non-transduced cells. EA-induced actin recruitment and internalization were assessed by live time-lapse confocal microscopy in non-transduced cells as a control group. In this group, EA (arrowheads) attaches, induces actin polymerization and invades (at 8 minutes frame) these cells after a short period of interaction. LifeAct-RFP (red) plasmid transfection was used to track filamentous actin, and the EA nucleus and kinetoplast were stained with Hoechst (white). These results are representative observations from at least 50 interactions of 2 independent experiments.

**Video 7.** Transduction and establishment of depleted cell lines do not interfere with actin recruitment and internalization of EAs by HeLa cells. EA-induced actin recruitment and internalization were assessed by live time-lapse confocal microscopy in HeLa cells transduced with a scramble sequence. In this group, EA (arrowheads) attaches, induces actin polymerization and invades (at 8-minute frame) these cells after a short period of interaction. LifeAct-RFP (red) plasmid transfection was used to track filamentous actin, and the EA nucleus and kinetoplast were stained with Hoechst (white). These results are representative observations from at least 50 interactions of 2 independent experiments.

**Video 8.** Depletion of N-WASP delays EA internalization. EA-induced actin recruitment and internalization were assessed in the indicated depleted lines by live time-lapse confocal microscopy. In N-WASP depleted cells, EA (arrowheads) attaches and induces actin polymerization but fails to invade these cells after interaction times similar to the control groups (between 30 and 40 minutes). LifeAct-RFP (red) plasmid transfection was used to track filamentous actin, and the EA nucleus and kinetoplast were stained with Hoechst (white). These results are representative observations from at least 50 interactions of 2 independent experiments.

**Video 9.** Depletion of WAVE2 delays EA internalization. EA-induced actin recruitment and internalization were assessed in the indicated depleted cell lines by live time-lapse confocal microscopy. In WAVE2 depleted cells, EA (arrowheads) attaches and induces actin polymerization but fails to invade these cells after interaction times similar to the control groups (between 30 or 40 minutes). LifeAct-RFP (red) plasmid transfection was used to track filamentous actin, and the EA nucleus and kinetoplast were stained with Hoechst (white). These results are representative observations from at least 50 interactions of 2 independent experiments.

# Supplementary Figures and Tables

## Supplementary Figures

**SUPPLEMENTAL FIGURE LEGENDS**


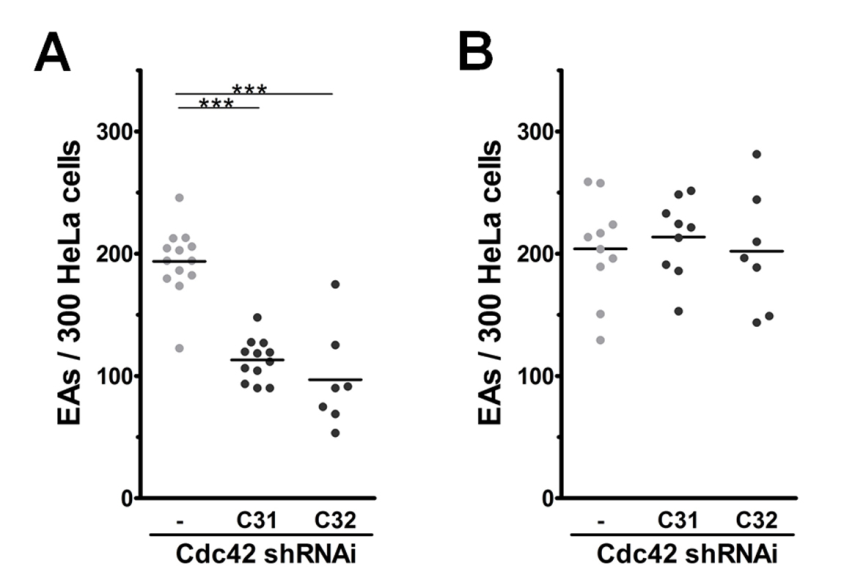


**Figure S1.** The role of Cdc42 in EA internalization was inconclusive based on shRNAi transduced cells. HeLa cells depleted of Cdc42 were incubated with EA for 2 hours, fixed with Bouin, and stained with Giemsa, and internalized parasites were quantified by optical microscopy. EA internalization was inhibited in some experiments (**A**, n ≥ 7) but not in others (**B**, n ≥ 7). The results represent the mean of at least three independent experiments in triplicate, and each dot represents one replicate (*** = P < 0.001).

**

**

**Figure S2.** Cells depleted of Rac1 present increased intensity and well-defined actin staining at EA invasion sites. Rac1 depleted and non-transduced cells were incubated with EAs for 120 minutes, and actin recruitment to EA invasion sites was evaluated by epifluorescence microscopy. EA-induced actin recruitment intensity is increased in cells depleted for Rac1 compared to the levels observed in the control groups ( - ). Actin (red), EAs (green) and DAPI (blue) were used to stain nuclei and kinetoplasts. Bars = 20 µm.





**Figure S3.** Depletion of host Rho-family GTPases, N-WASP or WAVE2 does not interfere with EA ability to recruit HeLa cell surface microvilli. Rac1 (R75), Cdc42 (C31), RhoA (R11), N-WASP (N61) or WAVE2 (W27) depleted cell lineages were incubated with EAs for 2 hours and washed, fixed and prepared for scanning electron microscopy as described in Materials and Methods. No significant changes were observed in morphology or the density of surface microvilli mobilized by EA (arrowheads) in depleted cells when compared to the non-transduced control group. Bars = 5 µm.


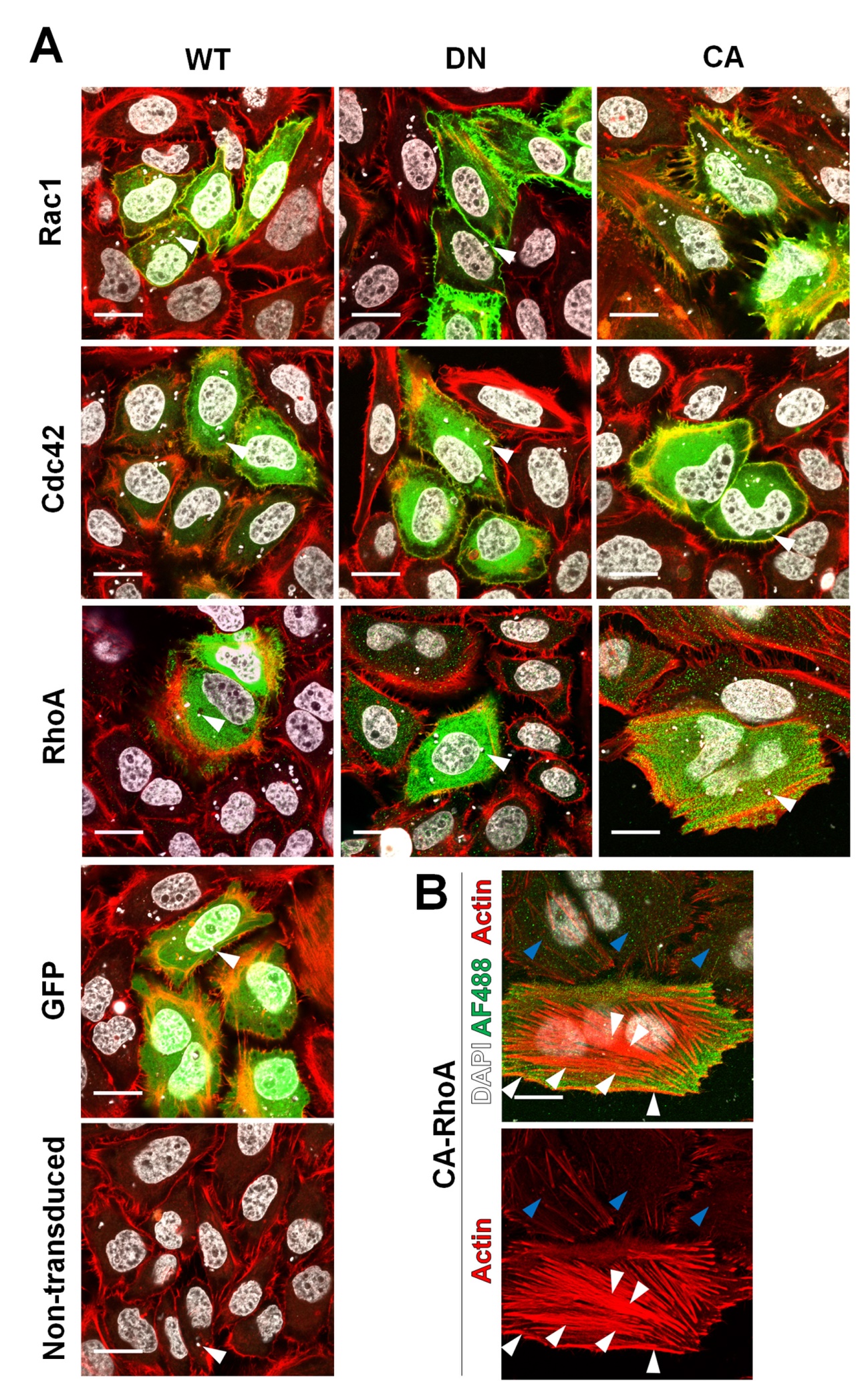


**Figure S4.** EA internalization is altered by the expression of activation mutated Rho GTPases. HeLa cells transfected with WT, DN or CA mutated GTPases were incubated with EAs (MOI 10) for 2 hours, and intracellular parasites were quantified under epifluorescence microscope. **(A)** Representative images showing internalized EAs (arrowheads) in transfected cells of the indicated groups. When compared to the GFP control group, the following observations were made: increased internalization of EAs in CA-Rac1 and WT-Cdc42; and reduced internalization of EA in DN-Rac1, CA-Cdc42, WT-RhoA and CA-RhoA. Green: GFP tagged (Rac1 and Cdc42) or Alexa Fluor 488 tagged constructs (RhoA-His); red: F-actin (phalloidin-TRITC); and white: nuclei and kinetoplasts (DAPI). Single focal plane images of cells infected with similar quantities of parasites as quantified in invasion assays (Fig. 5). **(B)** Cells overexpressing CA-RhoA display increased density and thickness of stress fibers (white arrowheads) compared to non-transfected cells (blue arrowheads). Green: RhoA-c-myc (Alexa Flour 488); red: F-actin (phalloidin-TRITC); white: cell nuclei (DAPI). Single focal plane images from the basal cell surface. Bars = 20 µm.
